# Better Teaching in the Schools

**Published:** 1921-08-20

**Authors:** 


### THE MATRONS' AND SISTERS' DEPARTMENT.

### BETTER TEACHING IN THE SCHOOLS.

The Nightingale Training School acts as a kind of barometer as regards the supply of candidates for the nursing service. In years when this school experienced a shortage of good entrants every hospital in the kingdom suffered more or less severely also. A good year at the Nightingale School forecasts improvement all round.

Happily the year 1920 saw the tide turn in the flow of women anxious to embrace the career of a nurse. The details furnished in the recently issued report of the Nightingale Fund clearly betoken a very marked improvement in the number of those applying and the calibre of those selected for the school. In 1919, we learn, difficulties were experienced in obtaining the right class of candidates for training. The numbers admitted to the school from the preliminary training-school fell to fifty-seven, and, unhappily, of these 30 per cent. resigned or were discharged as unsuitable. In 1920 a marked improvement took place; seventy-two probationers were admitted, and of these only twelve proved unsuitable. This improved state of affairs is paralleled in many other training-schools.

It has never been more important than at the present time that the standard of entrants should be raised, for it is not so much for the rank and file as for the women capable of directing others that the demand now exceeds the supply. The post of sister used in days gone by to be filled tolerably by very ordinary nurses, who passed on with such skill as they possessed the nursing lore they had themselves mastered, but were mainly concerned not so much with teaching as with getting the work of their wards accomplished. Under the new syllabus they will find themselves part of a great system, and the success or failure of the training-school to turn out acceptable candidates for examination will be found to rest on the ability of the ward sisters to give sound instruction.

It is sometimes feared that the appointment of a sister-tutor will tend to weaken the authority and responsibilities of the ward sisters. This seems, when it is fairly examined, to be a purely baseless fear. The standard of theoretical and practical training cannot fail to be sensibly raised under the influence of a wise sister-tutor charged with controlling the studies of the probationers. This must lead inevitably to a higher standard among the ward sisters. Everything they teach in their ward can be plainly seen to count. Probationers who are stimulated by intelligent tuition are themselves the greatest possible stimulus to their teachers, and the demonstrations carried out in the ward by the tutor cannot fail to illuminate the ward sisters as to the best methods for them to pursue. It was precisely in their teaching abilities that many ward sisters were weak, and this is not to be wondered at, for they had little, if any, formal instruction in the art. The sister-tutor's influence is likely to be powerfully felt in introducing improved methods to sisters who have had scanty opportunities of learning to make instruction clear to ignorant and puzzled probationers.

One after another hospital department becomes more highly organised and begins to demand the services of a highly trained sister capable of turning to good account all the teaching material available. At St. Thomas's three important posts were added last year:—"Sister Mothercraft," who supervises the ante-natal and child-welfare work in conjunction with the lady almoners and the out-patient departments; Sister District Midwife, whose sphere is the maternity district—a great asset to the maternity ward, which enables the hospital to take pupils in their fourth year for the prescribed term of four months' training; and Sister Venereal Department for Women. This last department has been much enlarged and developed, and now enables a four- or six-months' course, with lectures and instruction from the medical officer, to be given to nurses who have completed their training.

Thus side by side with the vital improvements in the general training of nurses special branches of nursing are being developed and a new set of specialists prepared for the public service.

